# A Spatial Division Clustering Method and Low Dimensional Feature Extraction Technique Based Indoor Positioning System

**DOI:** 10.3390/s140101850

**Published:** 2014-01-22

**Authors:** Yun Mo, Zhongzhao Zhang, Weixiao Meng, Lin Ma, Yao Wang

**Affiliations:** 1 Communication Research Center, School of Electronics Information Engineering, Harbin Institute of Technology, Harbin 150001, China; E-Mails: 11b905020@hit.edu.cn (Y.M.); wxmeng@hit.edu.cn (W.M); malin@hit.edu.cn (L.M.); wangyaowh2005@126.com (Y.W.); 2 Communication Department, Shenyang Artillery Academy, Shenyang 110867, China

**Keywords:** clustering, outliers, GA-SVM, kernel PCA, asymmetric matching

## Abstract

Indoor positioning systems based on the fingerprint method are widely used due to the large number of existing devices with a wide range of coverage. However, extensive positioning regions with a massive fingerprint database may cause high computational complexity and error margins, therefore clustering methods are widely applied as a solution. However, traditional clustering methods in positioning systems can only measure the similarity of the Received Signal Strength without being concerned with the continuity of physical coordinates. Besides, outage of access points could result in asymmetric matching problems which severely affect the fine positioning procedure. To solve these issues, in this paper we propose a positioning system based on the Spatial Division Clustering (SDC) method for clustering the fingerprint dataset subject to physical distance constraints. With the Genetic Algorithm and Support Vector Machine techniques, SDC can achieve higher coarse positioning accuracy than traditional clustering algorithms. In terms of fine localization, based on the Kernel Principal Component Analysis method, the proposed positioning system outperforms its counterparts based on other feature extraction methods in low dimensionality. Apart from balancing online matching computational burden, the new positioning system exhibits advantageous performance on radio map clustering, and also shows better robustness and adaptability in the asymmetric matching problem aspect.

## 1. Introduction

With the rapid development in the areas of mobile computing terminals and wireless techniques, indoor positioning systems have become unprecedentedly popular in recent years. Although the Global Positioning System (GPS) has been in service for decades, the indoor positioning ability of GPS is limited in indoor environments by the insufficient satellite coverage and poor positioning signals [1]. Not only does the indoor positioning draw attention from world famous academic research institutions but also large scale business activities have been deployed to solve this problem, such as the cooperation between Apple and WiFiSLAM, and the competition between Baidu and AutoNavi. As a consequence, several indoor positioning systems have been proposed in recent years, which are based on infrared [2], ultrasound and Radio Frequency (RF) [3], *etc.* Because the RF-based indoor positioning systems are capable of providing a wide range of coverage and using the existed WLANs as the fundamental infrastructure, fingerprinting methods [4–6] based on WLANs, as one of the most popular RF techniques, outperforms the other existing indoor positioning systems in civilian fields [7,8]. For instance, a convenient way based on propagation models for real-time indoor positioning without fingerprinting radio map basis is proposed in [9], but the Maximum Likelihood Estimation (MLE) and Least Square Optimization (LSO)-based probabilistic method used in the system would be time-consuming and computationally expensive in terms of mobile terminals. More importantly, the given confidence probability is lower than 10% under the condition that positioning accuracy is 2 m, which is sometimes insufficient for indoor positioning services, while fingerprinting positioning systems may normally provide confidence probabilities over 50% under the same conditions.

A typical fingerprinting indoor positioning system can be described as a situation where an end user takes RSS readings from available access points (AP) with a mobile terminal in an indoor environment. The positioning system then estimates the current location of the user according to a database, the so called fingerprint radio map, which contains pre-measured RSS values and the corresponding coordinates.

On the one hand, since a large indoor positioning region with a large fingerprint dataset could lead to high computational complexity and error margins, dividing it into several sub-regions is supposed to be able to improve the positioning performance [10]. Consequently clustering methods are widely applied to dividing the fingerprinting radio map into several sub-radio maps. However, the traditional clustering methods, e.g., K-Means, Fuzzy C-Means and Affinity Propagation [11,12], cannot theoretically process the outliers or singular points (an outlier means a sample point is assigned to a class by a cluster method but in physical space it is actually located in another class). This is a typical problem when deploying pattern recognition clustering methods in positioning systems. Most researchers simply ignore the outliers or delete those points, or artificially change the class label of the outlier to the one it is located in. Nevertheless, any of those solutions may lead to an increase in the positioning error rate. Furthermore, those methods for clustering the radio map essentially only depend on Received Signal Strength (RSS) values in signal space instead of considering their coordinate proximity in physical space. They actually generate the sub-radio maps in signal space, rather than in real sub-regions of the positioning area. Therefore, the coarse positioning in that case actually cannot prove that the terminal is located in a certain area, but only illustrate that the received RSS value may belong to one of the sub-datasets.

Besides, location privacy also should be taken into consideration sometimes [13]. For security reasons, sample points of certain areas such as confidential rooms within the radio map might be required to be clustered together, thereby providing the indoor positioning services of the dedicated area only to those authorized people. In this case, the traditional methods may not run well.

On the other hand, the deployment of feature extraction algorithms in the fingerprinting system is able to effectively process the radio map, *i.e.*, mapping it from the original signal space to a new feature space, thereby decreasing the noise interference and improving the location performance at the cost of increased computational complexity [14,15]. For instance, Reference [16] presents a positioning system based on Multiple (Linear) Discrimination Analysis (MDA or LDA) and Adaptive Neural Network (ANN). Though the Artificial Neural Network may suffer from the local minimum problem and over-fitting problems, the conception of Discriminant Components (DC) derived from MDA is efficiently introduced into the fingerprinting system. Parallel with DC, Principal Components (PC) derived from PCA is introduced in [17]. Apart from improved positioning accuracy, the proposed method also could reduce the number of training samples needed. Like the DC and PC used in [16–18], we pay attention to the aspect of dimensional reduction [19,20] (the original dimensionality of the radio map could be considered as the number of available APs) which is also a key factor for adjusting the available features of the feature extraction algorithm for indoor positioning. In fact, an appropriate algorithm can also enhance the robustness, balance the computational burden and save storage, which are all significant in terms of mobile computing.

Moreover, the number of APs received by a user in real-time phase may not always match the pre-stored radio map, e.g., one of those APs might be out of service or powered off at times. In that case, the traditional fingerprinting location method may not work out. Although some candidate options could deal with that, for instance set the RSS readings of the blocked AP as zero or remove the corresponding dimension of the radio map, the asymmetric matching problem still introduces severe systematic errors and reduces the positioning performance. However, by deploying an adaptive dimensional reduction technique, the impact of the missing APs could be strictly confined.

In this paper, for one thing, we propose the Spatial Division Clustering (SDC) method for reasonably dividing the radio map without singular points and the constraints presented above. After being integrated with optimized Support Vector Machine (SVM) technique [21,22], it is able to localize the test point (TP) into the sub region correctly during the so called coarse positioning process. To be specific, the SVM within the proposed positioning system is further optimized by a Genetic Algorithm (GA) [23], and generalized for multi-classification by the One *versus* One procedure. The proposed One *versus* One GA-SVM (OG-SVM) algorithm combined with the SDC method can reasonably cluster the radio map on the basis of coordinates and then classify the RSS sample into sub- regions for coarse positioning.

For another thing, we propose the Kernel PCA feature extraction algorithm based on Principal Component Analysis (PCA) [24–26] for dimensional reduction also as a solution for the asymmetric matching problem. Compared with other typical feature extraction methods such as Linear Discrimination Analysis (LDA) [27,28] and Local Discriminant Embedding (LDE) [29,30] used in positioning systems in our early works [14,15,20], the proposed method performs better in both low dimensional feature extraction and asymmetric matching accuracy when there is an AP outage.

The rest of this paper is arranged as follows: In Section 2, we will describe the structure of the traditional fingerprinting method for indoor positioning. After that, Section 3 starts with the introduction of the proposed new indoor positioning system, followed then by the theoretical analysis of the proposed SDC method with OG-SVM classification procedure and the Kernel PCA feature extraction method. In Section 4 we will provide experimental performances of the proposed methods and make comparisons with other typical algorithms. Section 5 finally presents the conclusions.

## 2. Fingerprinting Indoor Positioning System

A typical fingerprinting indoor positioning system is introduced in this section. Firstly, an end user takes RSS readings from available APs with his/her (WLAN adapter equipped) device in an indoor environment. The positioning system then estimates the current location of the user based on the measured RSS values by matching the received values with the fingerprint database, which is the pre-stored table of RSS values over a grid of reference points (both their RSS values and location coordinates are recorded) on the positioning area. Therefore the traditional fingerprinting method mainly consists of two parts, which are radio map building and the online matching procedures, respectively.

### 2.1. Source of Received Signal Strength

It is significant and necessary to briefly introduce where and how the RSS derives, based on which we could better analyze the unstable factors and sources of noise for the radio map. Actually, the RSS values derived from different APs are mainly calculated based on the received beacon frames of the device.

**Figure 1.**
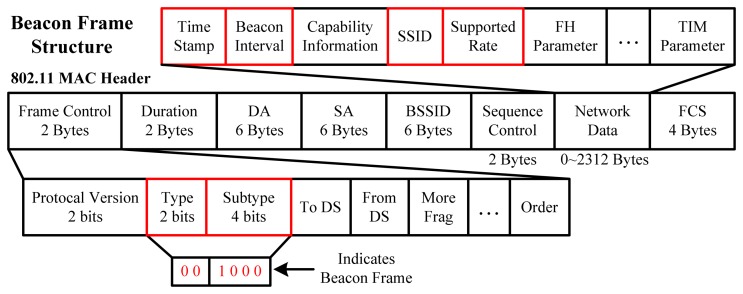
Main structure of a Beacon Frame.

The beacon frame is one of the management frames in IEEE 802.11-based WLANs and its structure is illustrated in Figure 1. It is periodically broadcast and terminal devices in passive scan mode can receive it without building a connection with any AP. The beacon frame is transmitted to announce the presence of a WLAN and includes all supported parameters. After receiving it, according to the information labeled with red rectangles in Figure 1, the terminal device is able to discriminate APs and calculate the RSS values over a sampling period. Specifically, The Beacon Interval is generally set to 100 microseconds; SSID identifies a specific WLAN; Supported Rate is a constant 1 Mbps and Time Stamp normally is used for compensation of interval inaccuracy [31]. Besides, the size of a beacon frame varies, depending on the instant transmitting status. Apart from the parameters presented above and the complexity of indoor propagation, the state of being in connection with an AP or not, the WLAN card, antenna and driver version of a terminal device (sensitivity of the adapter and the manufacturer) [32] also affect RSS values.

### 2.2. Building Radio Map

Radio map actually is a dataset used to bridge RSS values with location information. By setting amounts of Reference Points (RP), it is able to statistically describe the electromagnetic environment of an indoor positioning area. It is similar to many published researches [12,33] about fingerprinting where building a radio map is composed of two parts, which are sampling RSS values and recording coordinates information, respectively.

Firstly, we sample and record RSS readings at known locations with a mobile terminal device. As presented above, the height and the direction of a device antenna affects the online signals quality which directly influences the system positioning accuracy. For simplicity and concentrating on the proposed algorithms, as a compromise resolution, we only take a holding-in-hand situation (a user is holding the mobile in hand for using the positioning service, therefore the height of the terminal normally is set to 1.2 m) into consideration and take four RPs in four directions (North, South, East and West), respectively, from the same location (the four RPs in four directions share the same coordinates). We denote the RSS values derived from AP*i* at RP*j* as *ϕ_i_*_,_*_j_(δ)*, *δ* = 1,2,…,*q*, *q* ≥ 1 where *q* stands for the number of collected time samples, the average of the time samples thereby can be computed by:

(1) $$\phi_{i,j}=\frac{1}{q}\sum_{1}^{q}\phi_{i,j}\left(\delta \right)$$

where *ϕ_i,j_* is considered as actual RSS readings (in dBm) of AP*i* at RP*j*. So the radio map of RSS part is denoted as **Φ**:

(2) $$\Phi =\left[\begin{array}{cccc} \phi_{1,1} & \phi_{1,2} & \ldots & \phi_{1,M}\\ \phi_{2,1} & \phi_{2,2} & \ldots & \phi_{2,M}\\ \vdots & \vdots & \ldots & \vdots \\ \phi_{N,1} & \phi_{N,2} & \ldots & \phi_{N,M} \end{array}\right]$$

where *M* and *N* stand for the total number of available APs and RPs respectively. Therefore each row of **Φ**, the vector of the matrix, actually represents the RSS values of each RP, which is denoted as:

(3) $$\phi_{j}=\left[\phi_{j,1},\phi_{j,2},\phi_{j,3},\ldots ,\phi_{j,M}\right],\;j=1,2,\ldots ,N$$

Then, the radio map can be denoted as (*P_xy_^j^*, *ϕ_j_*), *j* = 1,2…*N*, *ϕ_j_* ∈ ℝ*^M^*, where the element *P_xy_^j^* is the coordinates of the RP*j*, which is represented by (*x_j_*, *y_j_*). In the case when no RSS readings can be detected from several APs at some RPs, the corresponding value is then set to be a minimal value instead of putting a zero because of the subsequent algorithm computation.

In addition, RSS should be collected systematically during different months or seasons which may cause evident RSS fluctuations. In this case, we could improve the system performance by enabling the radio map to store RSS samples of different periods and choose the corresponding database for the online matching process according to the current time which can be obtained from the timestamp of the beacon frame. Also, some extended Location Based Services (LBS) based on user gestures could be discriminated by built-in sensors of the mobile terminal firstly, and then the dedicated radio maps could be selected accordingly to provide the relative services.

### 2.3. WKNN for Online Matching

Many algorithms are widely used in fingerprinting method for matching the test points (TP) with the radio map, including *K*-Nearest Neighbors (KNN), Kernel Method [34], probabilistic approach [35] and Support Vector Regression (SVR) [15]. However, for simplicity and low complexity, we here take Weight *K*-Nearest Neighbors (WKNN) algorithm for the matching process in the proposed positioning system.

Specifically, in the online phase, a group of RSS readings is sampled by a terminal, and then it is matched with the most likely location by traversing all RPs of the radio map. For measuring the similarity between TP and each RP, WKNN algorithm calculates the distances between the TP and each RP by:

(4) $$D_{i}={\left(\sum_{j=1}^{M}{\left\|\phi_{\text{test},j}-\phi_{i,j}\right\|}^{p}\right)}^{\frac{1}{p}},i=1,2,\ldots ,N,j=1,2,\ldots ,M$$

where *ϕ_test,j_* is the received RSS value from AP *j* of TP, *D_i_* is the Manhattan distance and Euclidean distance when *p*=1 and 2, respectively. The first *K* RPs with the shortest distance are chosen to estimate the location of TP. Then the weight for each RP based on distance is defined as:

(5) $$\omega_{\zeta }=\frac{\sigma }{D_{\zeta }+\mu },\text{s}.\text{t}.\sum_{\zeta =1}^{K}\omega_{\zeta }=1,\zeta =1,2,\ldots ,K$$

where is the normalized parameter of the weight, *μ* is a minimal value set to prevent denominator becomes zero. Finally the output coordinates of the TP can be given by:

(6) $${P_{xy}}^{\text{test}}=\sum_{\zeta =1}^{K}\omega_{\zeta }{P_{xy}}^{\zeta },\zeta =1,2,\ldots ,K$$

It is obvious that the dimensionality of a radio map depends on both the number of RPs and quantity of deployed APs. Therefore, in the case of positioning a quite large area with many RPs needs to be set and numerous APs are required for dense coverage, so the size of radio map will be expanded considerably and the computational burden will be increased sharply. Besides, in case of some APs are broken down, the fingerprinting system may be severely damaged or even malfunction due to the missed dimension.

## 3. New Indoor Positioning System and the Proposed Methods Analysis

**Figure 2.**
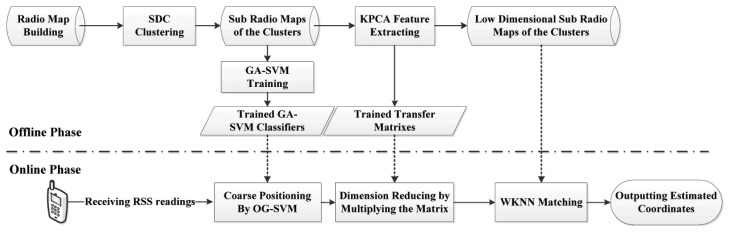
Flow chart of the proposed indoor positioning system.

The process used by some positioning systems is designed to transmit the RSS to a central server first for subsequent computing and then download coordinates from the server [16]. Different from that, the proposed system is designed to be able to run independently on a mobile terminal without a requirement of being in connection with any AP. But in this case, the trained radio maps and models need to be stored on the mobile terminal. For the purpose of reducing the fingerprint dataset thereby facilitating the mobile terminal resource consumption and improving robustness, the proposed positioning system is designed with two phases, which are the offline and online phase, respectively, and the corresponding flow chart is presented in Figure 2.

In the offline phase, RSS values are collected evenly on a grid with their coordinates as the radio map of the positioning area. After that the radio map is split into several sub-radio maps based on the SDC method. Then those sub-radio maps are trained by GA-SVM for building the classifiers. Thereafter the Kernel PCA algorithm is applied in each sub-radio map to extract the fingerprinting database into feature space and reduce the dimension of the radio maps. The low dimensional sub-radio maps for each cluster and corresponding trained transfer matrixes derived from the last step would be saved together with the GA-SVM classifiers and transferred to the mobile terminal for online real-time localization.

In the online phase, for real-time positioning, RSS values are measured by the mobile terminal user first. GA-SVM classifiers then will be used for locating the RSS value in the sub-region, which is also known as coarse positioning. Then, the transfer matrix of the sub-region is deployed to transfer the original received RSS values into corresponding low dimensionality in order to match with the low dimensional radio map of the sub-region. Afterwards, the WKNN algorithm is implemented as the precise location estimation method to match the RSS values with the low dimensional sub-radio map. Finally the positioning system outputs the estimated location coordinates.

**Figure 3.**
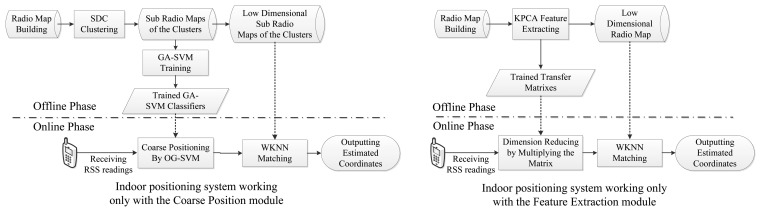
Flow charts of the indoor positioning system with a single module.

Moreover, it is worth noting that the computational complexity, positioning error rate and the resource limitations of mobile phones are all comprehensively considered in our proposed system. Therefore most of the computational consumption is handled in the offline phase by a powerful computer processor (*i.e.*, clustering sub-radio maps, training SVM classifiers and generating transfer matrixes), thereby relieving the computational burden introduced by the proposed algorithms in the online stage. Furthermore, the proposed new indoor positioning system is designed to be well modularized for conveniently adding other functionality modules. For instance, we could independently deploy the SDC with a OG-SVM coarse positioning module or Kernel PCA feature extraction module as two positioning systems, which are shown in Figure 3.

### 3.1. Spatial Division Clustering Method

As presented before, the outliers problem severely influences the coarse positioning accuracy and the integrity of sub-regions. Generally, the outliers only account for a small part of the radio map, but for a large scale radio map, getting rid of all the outliers may not be a reasonable way to proceed. Also, simply changing the class of those outliers to the nearest one may introduce unexpected errors, because, in terms of traditional cluster methods such as K-Means, the cluster centers would be changed accordingly as well.

The proposed SDC algorithm solves the problem by extracting the problem as a clustering process with distance constraints of physical location coordinates. The spatial division algorithm starts with defining the within-class scatter as:

(7) $${S_{w}}^{c}=\sum_{i=1}^{U}\frac{1}{U}\left(\phi_{i}^{c}-{\bar{\phi }}_{c}\right){\left(\phi_{i}^{c}-{\bar{\phi }}_{c}\right)}^{T},i=1,2,\ldots ,U,c=1,2,\ldots ,G$$

where *S_w_^c^* stands for the within-class scatter of the cluster *c*, and *c* ≤ *G* where *G* is the total number of possible clusters *U*, *U* ≤ *N* is the total number of RPs that belongs to the cluster *c*. $\phi_{i}^{c}$ are those vectors (RSS values) of the RPs within the cluster *c*, and *ϕ̄_c_* is the mean value of the counterpart, which can be given by:

(8) $${\bar{\phi }}_{c}=\frac{1}{U}\sum_{i=1}^{U}\phi_{i}^{c}$$

After that the between-class scatter is defined as:

(9) $$\begin{array}{cc} {S_{B}}^{c}=\frac{1}{G}\overset{G}{\underset{j=1}{\text{∑}}}\left({\bar{\phi }}_{c}-{\bar{\phi }}_{j}\right){\left({\bar{\phi }}_{c}-{\bar{\phi }}_{j}\right)}^{T}, & c=1,2,\ldots ,G,c\neq \end{array}j$$

where $S_{B}^{c}$ stands for the Between-class scatter of the cluster *c*, and *ϕ̄_c_* is the mean value of the RPs within the cluster *j*. Actually, *S_w_^c^* is the covariance matrix of the zero mean vectors assigned to the cluster *c* while the *S_B_^c^* is the covariance matrix of the cluster means, and the purpose of the proposed clustering algorithm is to optimize the radio between the within-class scatter *S_w_* and the between-class scatter *S_B_* which is denoted as *Q*, hence the objective function can be expressed as:

(10) $$\text{argmin}\overset{G}{\underset{c=1}{\text{∑}}}Q_{c}=\text{argmin}\overset{G}{\underset{c=1}{\text{∑}}}\frac{{S_{w}}^{c}}{{S_{B}}^{c}}$$

The definitions of the within-class scatter and the between-class scatter are primarily derived from the Fisher Criterion which is used in LDA. The proposed clustering algorithm for indoor positioning employs the minimum radio between *S_w_* and *S_B_* as the criterion mainly because of the fact that the RPs nearby each other would share the same spatial structure, which means that RPs within same class are supposed to be nearby each other and a within-class scatter should be as small as possible, while on the contrary RPs in different classes are supposed to be far away from each other and the between-class scatters should be as large as possible.

Therefore maximizing the similarity meanwhile minimizing the difference may effectively cluster the RPs. Different from the traditional clustering methods, taking *S_w_*/ *S_B_* as the measurement not only considers the distance between the independent RPs and updating the coefficient or cluster center, but also takes the similarity between classes into account. Instead of maximizing the value of the radio *Q* with classic convex optimization methods, the proposed algorithm previously assigns each two continuous RPs as a minimum class. It takes *Q* as the property of each class and runs clustering procedures in four steps as follows.

Step 1: Clustering centers determination
  The radio *Q* of each class can be computed by:
  (11) $$Q_{c}=\frac{{S_{W}}^{c}}{{S_{B}}^{c}}=\frac{G\overset{U}{\underset{i=1}{\text{∑}}}\left(\phi_{i}^{c}-{\bar{\phi }}_{c}\right){\left(\phi_{i}^{c}-{\bar{\phi }}_{c}\right)}^{\text{T}}}{U\overset{U}{\underset{J=1}{\text{∑}}}\left({\bar{\phi }}_{c}-{\bar{\phi }}_{j}\right){\left({\bar{\phi }}_{c}-{\bar{\phi }}_{j}\right)}^{\text{T}}}$$
  where *G* here equals to *N*/2 (in case of *N* is not divisible by 2, *G* equals to (*N* − 1)/2 and the last 3 RPs assigned to a class). Then calculating the similarity of each pair of *Q*, hence the similarity between one class and all others is referred to as:
  (12) $$\begin{array}{cc} {S_{Q}}^{c}=\overset{G}{\underset{i=1}{\text{∑}}}\left\|Q_{c}-Q_{i}\right\|, & c=1,2,\ldots ,G,i\neq c \end{array}$$
  The *Q* of class *c* corresponding to the *max S_Q_^c^* is chosen as the first cluster center which is denoted as *Ctr*^1^. Then we compare all the other *Q* with the *Ctr*^1^ and find the one with the lowest similarity (*i.e.*, to find *max* ‖*Q_c_* − *Ctr*^1^‖, *c* = 1,2, …, *G*) as the second cluster center *Ctr*^2^. For the third center and so on, the similarity is calculated in advance, namely:
  (13) $${S_{Q}}^{ij}=\left\|Q_{i}-Ctr^{j}\right\|,i=1,2,\cdots ,G,j=1,2,\cdots ,E$$
  where *E* is the number of centers have been set. Therefore the next most suitable center with the least similarity can be set by min *S_Q_^i^*^,^*^j^*, hence the (*E* + 1)th center is the *Q* of class *i* subjected to *max*{*min*(*S_Q_^i^*^,1^, *S_Q_^i^*^,2^, …, *S_Q_^i^*^,^*^E^*)}. *i* = 1,2, …, *G*.
Step 2: Combination of clusters
  Based on the centers derived from the previous step, the following process is to calculate similarity between each class and its centers, where Equation (13) is deployed here. Then the class is assigned to the most similar center in turn. Meanwhile, *Q* of the center will be updated by Equation (11) after each class is allocated in. If the total number of centers *E* is assigned, then *E* clusters will be formed consequently.
Step 3: Splitting of the clusters
  In order to meet the condition that no outliers in positioning area after the radio map is clustered, RPs within a class is supposed to be subjected to the criterion:
  (14) $$\sqrt{{\left(x_{i}-x_{j}\right)}^{2}+{\left(y_{i}-y_{j}\right)}^{2}}\leq \varepsilon$$
  where *x_i_y_i_*, *x_j_y_j_* are any two sets of coordinates of RPs within a same class, and *ε* is the distance threshold based on the density of sampled RPs and location environment. Different from the combination process based on the signal features, the splitting process depends on the coordinates information (which is another part of radio map), namely:
  (15) $$\text{Loc}=\left({{\text{P}}_{xy}}^{1},{{\text{P}}_{xy}}^{2},\cdots ,{{\text{P}}_{xy}}^{N}\right)$$
  Denoting the coordinates information of cluster C as:
  (16) $${\text{Loc}}^{\text{C}}=\left({{\text{P}}_{xy}}^{\text{C}1},{{\text{P}}_{xy}}^{\text{C}2},\cdots ,{{\text{P}}_{xy}}^{\text{C}U}\right)$$
  where P*_xy_^C^* stands for the coordinates information of the RPs belonging to the cluster C, and *U* here is the total number of RPs belong to the cluster C. Then the procedures of cluster splitting are addressed as follows:
  - **Initialization**: Initialize the P*_xy_^C^*^1^ as an element of new cluster C_1_, where C_1_ is considered as the first sub cluster of C.
  - **IF** P*_xy_^C^*^2^ satisfy the criterion Equation (14) with P*_xy_^C^*^1^, **THEN** assign it to C_1_.
    **ELSE** set the P*_xy_^C^*^2^ as an element belongs to a new cluster C_2_.
    **End IF**
  - **FOR** P*_xy_^Ci^*, *i* = 3,4, …, *U*
    **IF** P*_xy_^Ci^* meets the criterion Equation (14) with P*_xy_^Cj^*, *j* = 1,2, …, *i* − 2, **THEN** assign P*_xy_^Ci^* to the cluster which P*_xy_^Cj^* belongs to.
    **ElSE IF** P*_xy_^Ci^* meets criterion Equation (14) with more than one P*_xy_^Cj^*, **THEN** combine the clusters corresponding to those P*_xy_^Cj^* with the P*_xy_^Ci^* as a new cluster. P*_xy_^Ci^* works as bridge connection.
    **ELSE** set a new cluster with P*_xy_^Ci^* as an element.
    **END FOR**
  For special requirements of the indivisible sub region, we could assign the RPs within that region as an independent cluster without participating in the combination and splitting steps.
Step 4: Outputs of clustering
  Looping step1 to Step 3 until the number of output clusters comes to convergence, and then the clusters are formed. For some of the small clusters, they could be simply assigned into the nearest clusters. Finally the whole SDC method process is completed.

### 3.2. Classification by OG-SVM

#### 3.2.1. Introduction of SVM in the Positioning System

OG-SVM is deployed to distinguish the TP to which cluster it belongs to, and locate it in the sub-region for the coarse location process. An introduction to SVM deployment in positioning is briefly given first. Denoting (*ϕ_i_*,*L_i_*), *i* = 1,2, …,*N*, *ϕ_i_* ∈ ℝ*^M^* (according to the experimental positioning environment, *N* here is the total number of RPs of two clusters) as the set of training samples, where *ϕ_i_* is the vector of RP as mentioned before, and *L_i_* ∈ {1, −1} labels which class the vector belongs to. The purpose of SVM is to obtain the weight vector **w** and the scale *b*, such that:

(17) $$L_{i}\left(\langle w\cdot \phi_{i}\rangle +b\right)\geq 1$$

where 〈**w** · *ϕ_i_*〉 stands for the inner product of the vectors **w** and *ϕ_i_*. 〈**w** · *ϕ_i_*〉 + *b* is the so called hyper-plane that enables the training samples with the same label separate with others. In the case of nonlinear condition, a slack variable is introduced and denoted as *ζ_i_* ≥ 0, *i* = 1,2, …,*N*, so Equation (17) is converted to:

(18) $$L_{i}(\langle w\cdot \phi_{i}\rangle +b)\geq 1-\xi_{i}$$

The objective function is:

(19) $$\min\left(\frac{1}{2}\langle w\cdot w\rangle +C\sum_{i=1}^{N}\xi_{i}\right)$$

where *C* is the key penalty parameter and element $\sum_{i=1}^{N}\xi_{i}$ defines maximum number of training errors. Also the inner product 〈*ϕ_i_* · *ϕ_j_*〉 is replaced by kernel function, which is expressed as *K* 〈*ϕ_i_* · *ϕ_j_*〉. The kernel methods are able to map the nonlinear dataset into a high (even infinite) dimensional feature space from which the dataset could be linearly separable. Radial basis function (RBF) is one of the kernel methods and is adopted in the proposed positioning system, which is defined as:

(20) $$K\left(\phi_{i},\phi_{j}\right)=\exp\left\{-g{\left\|\phi_{j}-\phi_{i}\right\|}^{2}\right\}$$

where *g* is another key parameter geometrically defining the width of the RBF. This might lead to the over-fitting problem if *g* is relatively small, while on the contrary, the flexibility and robustness might be weakened.

Lastly, the decision function or so called SVM classifier of the indoor positioning system can be obtained as:

(21) $$f\left(x\right)=\text{sign}\left(\langle w^{*}\cdot \phi \rangle +b\right)$$

Where w* is the solution of the optimal separating hyper-plane (OSH) that enables the samples with different labels to be most distinguishable, *ϕ* is the vector of a test point with unknown class label, and the output of the function will decide which class it belongs to (positive result decides one class and negative output decides another one).

#### 3.2.2. Genetic Algorithm for SVM Optimization

Although SVM theoretically is a quadratic optimization problem and the optimal solution is given, the parameters *C* in Equation (19) and *g* in Equation (20) still need to be chosen properly due to reasons mentioned before. Therefore GA is integrated into the SVM training process to adjust the two parameters adaptively.

The Genetic Algorithm is derived from the bionic process in which a population evolves by competing with others and preserving its superiority in Nature. Each individual in a population would be eliminated for its weak adaptability or kept due to its strong performance. Consequently the new generation becomes more robust and adaptive.

GA is able to search a large solution space efficiently by adopting probabilistic transition procedure mechanics. It mainly includes three steps, which are selection, crossover and mutation. To be specific, selection is aimed at electing the optimal individuals for reproducing the next generation; Crossover is applied for exchanging information, thereby preserving and collecting the genetic advantage; Mutation is designed to introduce the variation for making new individuals. In terms of GA-SVM, the fitness function is defined as:

(22) $$\min F(C,g)=\frac{1}{1+\kappa }$$

where *κ* is the classification accuracy rate. The searching space of the parameter *g* is defined by min ‖*ϕ_j_* − *ϕ_i_*‖^2^ × 10^−3^, max ‖*ϕ_j_* − *ϕ_i_*‖^2^ × 10^−3^ while the counterpart of *C* is (0, 10). Generally, after randomly initializing the population, the fitness of each individual is calculated by Equation (22). Then a probability will be assigned to each individual according to the fitness (higher fitness value with higher probability). After that, new individuals are generated by the crossover and mutation operations. The whole process would be repeated until the new individual meets the preset values. Finally with *N*-fold cross validation (*i.e.*, training data is separated into *N* parts, one of which is deployed for validating accuracy while the remaining parts are the training sets, and the procedure is taken by *N* turns), the optimal combination of the parameters (C^*^, g^*^) can be obtained.

#### 3.2.3. OG-SVM Method

Due to the fact that generally more than two clusters (or sub-regions) exist within an indoor positioning area, One *versus* One GA-SVM is adopted as the classification algorithm to deal with the multiple classes. Instead of deploying a multiple-class SVM, the OG-SVM method sets a group of binary-class SVM classifiers optimized by GA to perform the classification. To be specific, supposing that there are *G* clusters in the positioning region, there are *G*(*G* − 1)/2 SVM classifiers that can be obtained after training each two clusters as a group with GA-SVM. In term of classifying a test point, it will be put into all SVM classifiers in turn. If it goes to the cluster *c*, *c* = 1,2,…,*G*, then cluster *c* gets 1 vote. Consequently the test point belongs to the cluster with most votes and thus the corresponding sub-region can be located.

### 3.3. Dimensionality Reduction by Kernel PCA

Kernel PCA is used in the proposed indoor positioning system to extract the features of the radio map and reduce its dimensionality. An analysis on Kernel PCA is presented below.

As denoted before in the proposed positioning system the RSS values of a cluster is given by **Φ***_c_* = {*ϕ_1_*,*ϕ_2_*,…,*ϕ_U_*}, where *U* is the total number of vectors belong to the cluster *c*. In order to meet the constraint of PCA, vectors of **Φ***_c_* has to be decentralized previously. Defining the nonlinear mapping *∂*: ℝ*^M^* → **
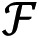
** where ℝ*^M^* is the Euclidian space of samples and **
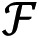
** is the feature space where inner product can be computed by a kernel function. Then the covariance matrix of the samples in feature space can be given by:

(23) $$\bar{C}=\frac{1}{U}\sum_{i=1}^{U}\partial \left(\phi_{i}\right)\partial {\left(\phi_{i}\right)}^{\text{T}}$$

Denoting *λ* and **v** as the eigenvalue and the eigenvector of *C̄* respectively, then the eigen-decomposition can be given as:

(24) $$\lambda V=\bar{C}V$$

Based on the fact that the eigenvector **v** can be expressed in linear spanning space of *∂*(*ϕ_i_*), *i* = 1,2, … *U*, namely:

(25) $$V=\sum_{i=1}^{U}\eta_{i}\partial (\phi_{i})$$

where *η_i_* is the weight coefficient for each *∂*(*ϕ_i_*), we could substitute Equation (25) into Equation (24) and by pre-multiplying *∂* (*ϕ̂_j_*)*^T^*, *j* = 1,2, … *U*, then the equation can be given as:

(26) $$\begin{array}{c} \lambda \left(\overset{U}{\underset{i=1}{\text{∑}}}\eta_{i}\partial {\left(\phi_{j}\right)}^{\text{T}}\partial \left(\phi_{i}\right)\right)=\partial {\left(\phi_{j}\right)}^{\text{T}}\cdot \frac{1}{U}\overset{U}{\underset{l=1}{\text{∑}}}\partial \left(\phi_{l}\right)\partial {\left(\phi_{l}\right)}^{\text{T}}\cdot \overset{U}{\underset{i=1}{\text{∑}}}\eta_{i}\partial \left(\phi_{i}\right)\\ \lambda \left(\overset{U}{\underset{i=1}{\text{∑}}}\eta_{i}K_{ji}\right)=\frac{1}{U}\overset{U}{\underset{l=1}{\text{∑}}}\overset{U}{\underset{i=1}{\text{∑}}}\eta_{i}K_{ji}K_{li} \end{array}$$

and the equation can be further expressed as $\lambda {\left(K\eta \right)}_{j}=\frac{1}{U}{\left(K^{2}\eta \right)}_{j}$, where **K** = [*K*(*ϕ_i_*, *ϕ_j_*)]*_U_*_×_*_U_*, **η** = (*η*_1_, *η*_2_, …, *η_n_*)^T^. Consequently it can be converted to:

(27) $$\hat{\lambda }\eta =K\eta$$

Where *λU* is substituted by *λ̂*. After eigen decomposition, denoting *λ^1^*, *λ^2^*, …,*λ^U^* are the eigenvalues and **η**^1^, **η**^2^, …, **η**^U^ are the eigenvectors of **K** respectively, therefore the *i-*th eigenvalue and eigenvector can be given by:

(28) $$\lambda_{i}=\frac{{\hat{\lambda }}^{i}}{U},V_{i}=\sum_{j=1}^{U}\eta_{j}^{i}\partial (\phi_{j})$$

where $\eta_{j}^{i}$ is the *j-*th element of **η***^i^*, *i* = 1,2,…,*U*. Hence, the projection of a test sample *ϕ* on *j*-th axis of the feature space is represented by:

(29) $$\partial {\left(\phi \right)}^{\text{T}}V_{j}=\Delta^{j}\sum_{i=1}^{U}\eta_{i}^{j}\partial {\left(\phi \right)}^{\text{T}}\partial (\phi_{i})=\Delta^{j}\sum_{i=1}^{U}\eta_{i}^{j}K(\phi ,\phi_{i})$$

where Δ*^i^* is a normalized factor computed by equation (**V**)^T^·**V** = 1. By adopting the maximum first *d* eigenvalues *λ̂*^1^,*λ̂*^2^,…,*λ̂^d^* and their corresponding *d* eigenvectors **η***^1^*,**η***^2^*,…,**η***^d^* where *d* ≪ *U*, the high dimensional dataset can be accordingly reduced to *d* dimension.

After defining the radio map of cluster *c* as $\Phi_{c}^{o}=(P_{xy}^{i},\phi_{i}^{o})$, *i* = 1,2, …, *U*, *ϕ_i_* ∈ ℝ*^M^* and its low dimensional counterpart as $\Phi_{c}^{o}=(P_{xy}^{i},\phi_{i}^{o})$, *i* = 1,2, …, *U*, $\phi_{i}^{o}\in {\mathbb{R}}^{d}$, the transfer matrix of the region can be expressed as:

(30) $$M=\left(\Delta^{j}\sum_{i=1}^{U}\eta_{i}^{j}K(\phi ,\phi_{i})\right),j=1,2,\cdots ,d$$

To conclude, in the offline phase of the positioning system, a low dimensional radio map for each cluster is generated by deploying the Kernel PCA algorithm with RBF aligned with the kernel function used in SVM. In the online phase, after a test point is located to a cluster by OG-SVM, the corresponding low dimensional radio map will be chosen accordingly. Therefore, a downsized test point after being decentralized can be computed by Equation (30) (*i.e.*, running Equation (29) *d* times for *d* axis or *d* dimensions) and expressed as $\phi_{i}^{o}=[\phi_{i,1},\phi_{i,2},\ldots ,\phi_{i,d},]$. Moreover, the transfer matrix could be integrated or further compressed by mathematic methods [36,37]. The WKNN algorithm will finally be deployed as the measuring method for matching the $\phi_{i}^{o}$ throughout the radio map $\Phi_{c}^{o}$ thereby obtaining the estimated coordinates.

## 4. Implementation and Performance Analysis

In general, the proposed indoor positioning system runs as following procedures: for the offline phase, firstly, we start by constructing the radio map. Secondly, we cluster it into several sub-radio maps by the SDC method. The third step is to train the sub-radio maps with OG-SVM, generating classifiers. Then, the following step is to reduce the dimension of each sub-radio map by Kernel PCA and generate the corresponding transform matrixes. For the online phase, firstly, we classify the test point to the sub-region by the OG-SVM method with those classifiers. After that the dimensions of the test point are reduced by the matrix generated offline. The final steps are matching the low dimensional test points with the low dimensional sub-radio maps by WKNN, and outputting the estimated coordinates. In this Section the experimental evaluations of the proposed method for indoor positioning system are elaborated in detail.

### 4.1. Indoor Positioning Environment

**Figure 4.**
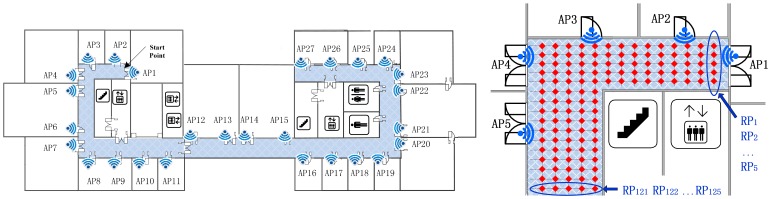
Floor plan for the indoor positioning experiment and reference point setting.

Figure 4 shows a floor plan of a research center. The fingerprint dataset was carefully measured in this typical office environment. The proposed indoor positioning system is built here with 27 Access Points (marked as AP1-AP27) located evenly in each room. Then we individually sample and record the RSS readings 100 times at each reference point (with a sampling rate of 2 times per second) with a mobile terminal. The area of interest colored with blue is the corridor part (49.4 m × 14.1 m), where 828 locations are equally distributed as the experimental RPs.

### 4.2. Cluster Performance of SDC Method

In this subsection, the proposed SDC method is evaluated well in terms of both radio map division and positioning accuracy for indoor localization. K-Means and Fuzzy C-Means (FCM) algorithms are also implemented for verifying the analysis and testing the performance by comparison.

**Figure 5.**
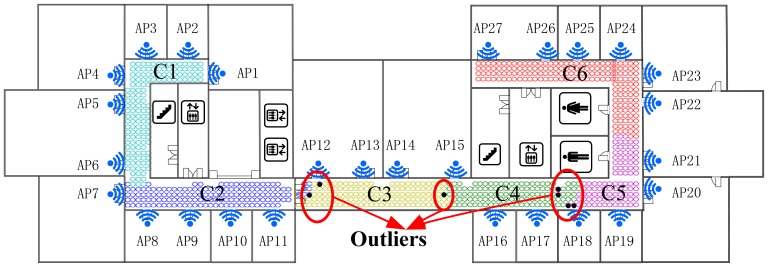
Positioning area clustered by the FCM algorithm.

**Figure 6.**
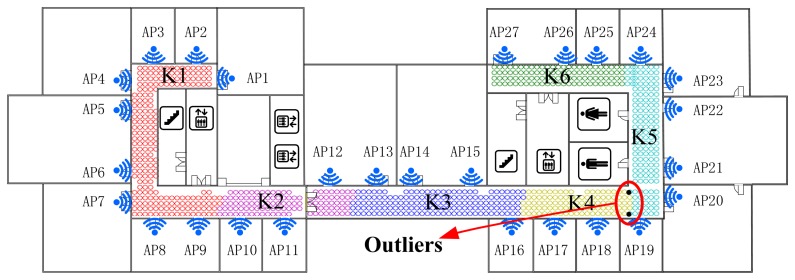
Positioning area clustered by the K-Means algorithm.

As shown in Figures 5 and 6, the Radio Map is clustered into six (marked as F1-F6 and K1-K6 respectively) sub-areas by deploying FCM and K-Means algorithm, where different colors represent different sub-regions and the black points stand for the outliers. In addition, the white blanks among the RPs are obstacles in the building where RSS cannot be tested.

**Figure 7.**
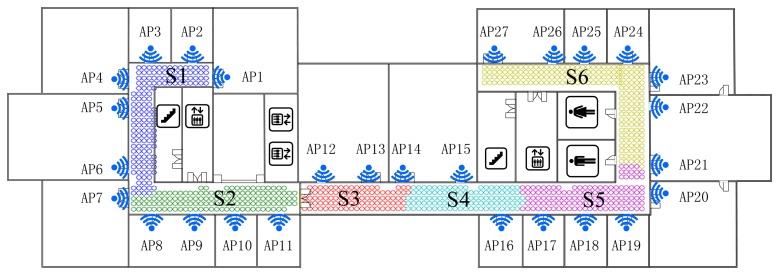
Positioning area clustered by the SDC algorithm.

This demonstrates that, for clustering using FCM, the radio map is divided almost symmetrically but the outliers are distributed mainly in the middle three clusters and account for nearly 1% (7/828) of RPs, while for K-Means clustering, the divided sub-regions are slightly unbalanced in term of RP quantity, but few outliers exist in those regions. It is worth noting that the RPs are sampled on the grid evenly, and the experimental environment is relatively stable (few people walk around and all windows are closed). In this case, the outliers are supposed to be far less than in a practical environment. The proposed SDC method divides the interesting area as illustrated in Figure 7, where different regions are marked as S1-S6 with different colors. Compared with the other two algorithms, the SDC method is able to cluster the RPs more symmetrically without any outliers problems.

Actually, dividing the radio map symmetrically may not prove that the clustering method is effective and suitable. Nevertheless, the structure of the experimental region is nearly balanced, building materials are almost uniform and all APs are arranged evenly. Therefore, in this case, clustering the RPs in a symmetric way is supposed to be more reasonable. Besides, the boundaries of each cluster are located near the corner or doors where RSS values normally fluctuate and are more distinguishable. It also demonstrates the reliability and effectiveness of the proposed SDC method based on the divided structure.

**Figure 8.**
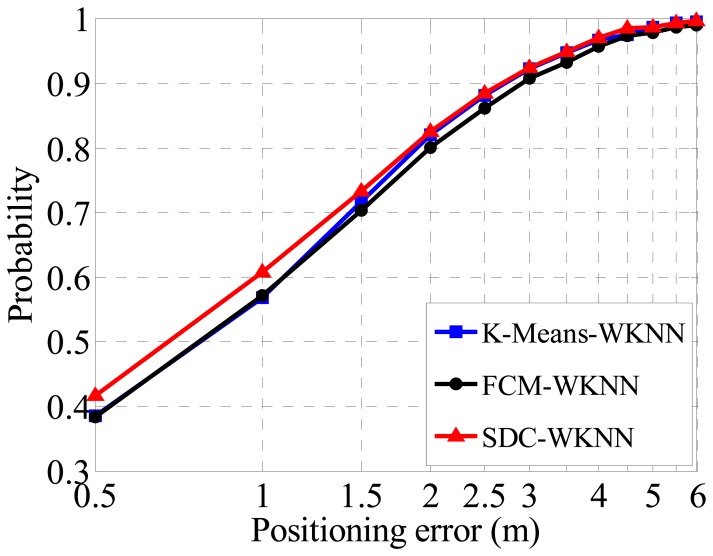
Positioning accuracies based on three different clustering methods.

In order to verify the performance of different clusters in term of positioning accuracy, the WKNN method is directly deployed to all divided sub-regions for fingerprint localization based on the three clustering cases without considering coarse positioning (*i.e.*, assuming that which sub-region a TP belongs to is known). The fine positioning accuracy is shown in Figure 8, where the FCM method achieves a Confidence Probability (CP) over 80% with a positioning error (PE) within 2 m. For the K-Means algorithm, the CP is 2% better than the counterpart of the FCM. It is notable that the positioning accuracies are calculated for each region independently, and then added together with weights of RPs numbers of a cluster. The performance of the proposed SDC method is the same as that of the K-Means as PE equals 2 m too, but it is slightly superior to other algorithms when the PE is 1 or 1.5 m. Therefore, the proposed SDC method is better than other clustering methods for indoor localization due to its better positioning performance.

### 4.3. Coarse Positioning Performance of the OG-SVM Method

**Figure 9.**
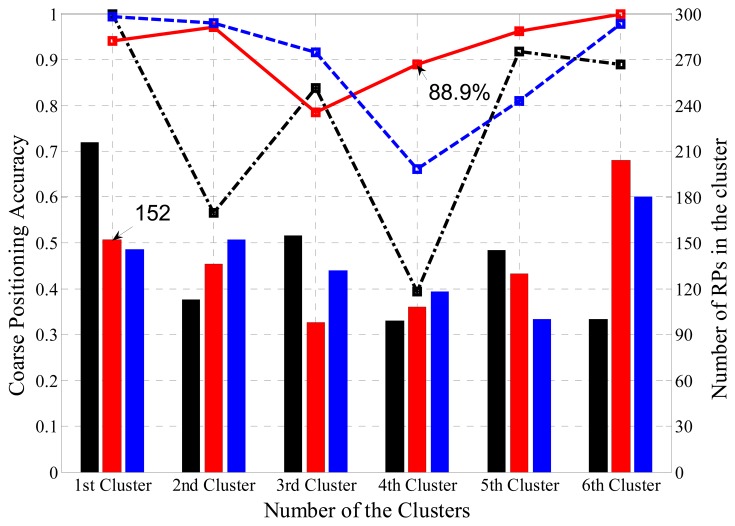
The different clustering results and the coarse positioning performances for the three methods.

Coarse positioning is responsible for allocating received RSS readings to the sub-regions where they belongs. The integrated information of the coarse position for the three clustering methods is demonstrated in Figure 9, where the black, red and blue bars represent the number of RPs in the regions clustered by K means, SDC and FCM, respectively, while the black, red and blue lines stand for the coarse positioning accuracies in the regions clustered by K Means, OG-SVM and FCM, respectively. For example, the first region (labeled as S1 before) clustered by SDC consists of 152 RPs, and OG-SVM coarse positioning accuracy of the S4 region is 88.9%. It clearly shows the distribution of RPs in all six regions and the classification accuracy for each cluster and each clustering method.

To be more specific, the coarse positioning accuracy based on the FCM algorithm for each cluster is listed in Table 1, while the coarse positioning accuracy of the K-Means algorithm is shown in Table 2. The overall classification (*i.e.*, coarse positioning) accuracy of FCM is about 10% higher than the K-Means (90.58% and 81.04%, respectively). Therefore, even if few outliers appear in the K-Means clusters which performs better than FCM, in terms of the coarse positioning accuracy it actually shows a reverse outcome.

Besides, both tables show that the coarse positioning accuracy of the first and the last clusters are much higher than the clusters in the middle. According to the experimental results and previous analysis of the RSS database, it can be deduced that classification criterion based on the cluster centers, which is used by FCM and K-Means, runs well in the areas with distinguishable RSS values, but may not classify the TPs efficiently in the regions where RSS change stably or fluctuate within a narrow range.

Compared with the two traditional clustering algorithms, K-Mean and FCM, the coarse positioning based on SDC with OG-SVM performs better, as shown in Table 3. Specifically, the classification accuracy of the proposed method is 93.84%, which is 12.80% greater than the result of K-Means and 3.26% higher than the FCM, while no outliers occur.

**Figure 10.**
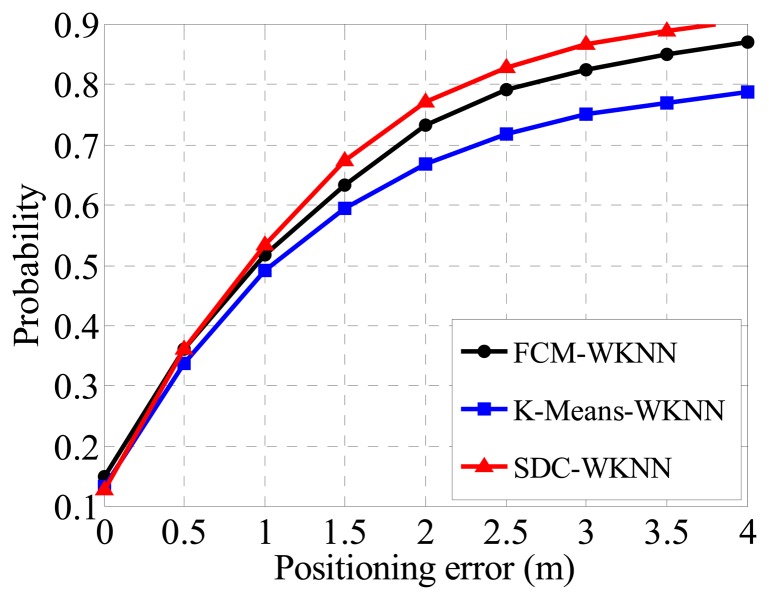
Positioning accuracies based on Coarse Positioning procedure.

Taking the coarse positioning procedure into the fingerprinting system (which actually is the single module system shown on the left of Figure 3), the advantage of the proposed SDC and OG-SVM method would be more apparent. As illustrated in Figure 10, the final estimated positioning accuracy of the proposed method is 77.4% under the condition that the positioning error is within 2 m. Compared with the 73.3% positioning accuracy of FCM and the 66.9% of K-Means under the same conditions, the proposed coarse positioning method is more effective and precise, thereby ensuring the following fine positioning procedure. Besides, according to extended experimental results, the coarse location accuracy of the proposed method can be further improved with more training samples in the OG-SVM, also clustering the radio map into a smaller number of regions by the proposed method may yield a better performance.

### 4.4. Low Dimensional Performance of Kernel PCA Method

**Figure 11.**
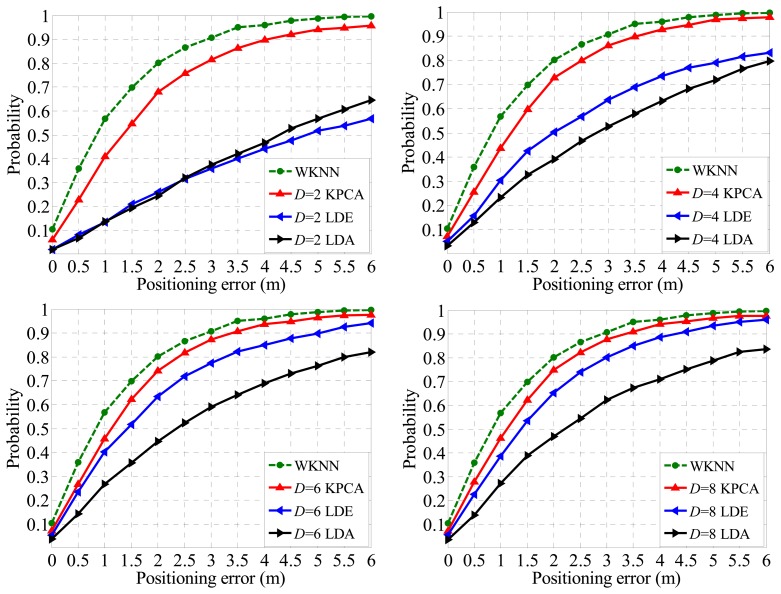
Positioning accuracies comparison between methods in the cases of *D* = 2, *D* = 4, *D* = 6 and *D* = 8, respectively.

Theoretically, feature extraction algorithms are able to improve the positioning accuracy by learning the inner structure of the dataset and eliminating part of the noises normally with a high dimension [14,15], but in this paper we focus on the capacities of different algorithms in very low dimensionality scenarios. As a direct evaluation of the low dimensionality performance of different feature extraction algorithms, Figure 11 demonstrates that the relationship between Confidence Probability (CP) and the Positioning Error (PE) distance. Specifically, the green dashed line represents the performance of the WKNN fingerprinting method with full dimensionality (27 dimensions for 27 APs), the red line stands for the performance of WKNN fingerprinting after dimensional reduction by the KPCA method. Similarly, the green and black lines represent the counterparte of the LDE and LDA methods, respectively.

As typical linear and manifold feature extraction methods, both LDE and LDA show significant properties in many pattern recognition aspects, however, in terms of extracting eigen-features within an indoor radio map, the Kernel PCA method reveals a better fitness, because of the fact that in the cases of *D* = 2, 4, 6, 8 where *D* stands for the dimensionality, the Kernel PCA method shows more outstanding performance according to the experimental result shown in Figure 11.

As shown in Figure 11, the WKNN method achieves a CP of about 80% under the condition that PE is within 2 m. Compared with other algorithms, along with the increasing dimensionality, CP of the Kernel PCA approaches the WKNN faster. Therefore the proposed method outperforms other algorithms in a low dimensionality situation. For example, the CPs of LDA and LDE are 39.2% and 50.1%, respectively, under the condition that *D* = 4 and PE is within 2 m. the performance of the proposed Kernel PCA reaches up to 72.5%, which is less than the dimension-unreduced WKNN method, but far more competitive than others. Moreover, in this case the size of the radio map for online matching process is reduced 85% (calculated by (1 − 4/27)).

In addition, the number of nearest neighbors *K* also affects the WKNN positioning accuracy in this situation. We set the optimized value of *K* as 4 based on experiments. It is also worth noting that the WKNN method is supposed to perform best in an ideal experimental environment (small noise intensity) because compared with other dimension-reduced methods, it works on full dimensionality with all the radio map information. Dimensionality reduction actually implies that part of the information has to be lost though a comprehensive preprocessing has been done before in the feature extraction procedure.

### 4.5. Asymmetric Matching of the Kernel PCA Method

**Figure 12.**
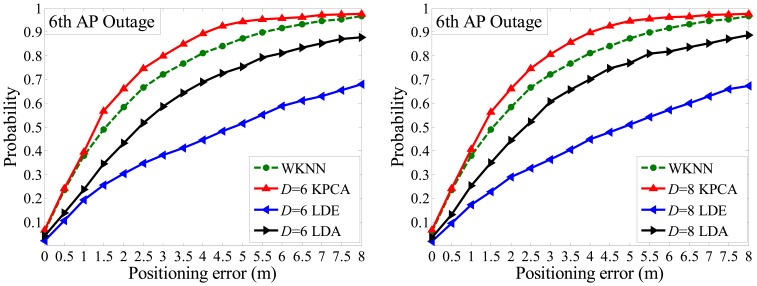
Positioning accuracies comparison when *D* = 6 and *D* = 8 respectively in the case of 6th AP outage.

**Figure 13.**
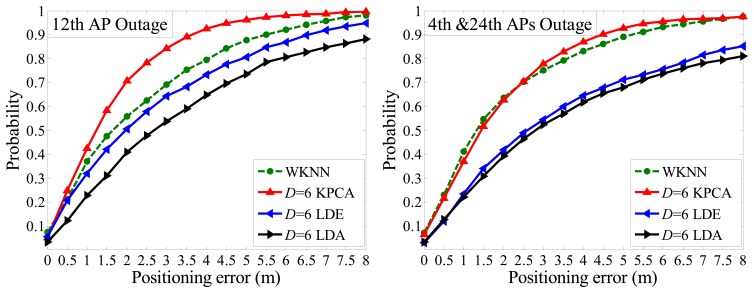
Positioning accuracies comparison in the cases of 12th AP outage and both 4th, 24th APs outage respectively.

It is unavoidable that outages might occur occasionally, in which case the WKNN fingerprinting method is drastically affected and even fails to work. Taking the WKNN method as experimental counterpart, we assign the missed dimension as a group of minimum value. Then, according to Figures 12 and 13 below, under the condition that PE is within 2 m, the CPs of the WKNN method are 58.3%, 56.8% and 64.4% when the 6th AP, 12th AP and both 4th 24th APs is/are powered off, respectively. Generally, CP declines sharply about 20% compared with the case that all APs run well.

However, the proposed Kernel PCA method is far less affected by AP outages than the WKNN and other methods. For instance, with the situation that *D* = 6 and PE is within 2 m, it only declines 4% of CP when the 6th AP is powered off. Also, it keeps CP over 60% in all three cases (6th AP outage, 12th AP outage, both 4th and 24th APs outage). Specifically, under the condition that *D* = 6 and PE is within 2 m, the CPs of Kernel PCA method are 66.3%, 71.5% and 62.5%, respectively, which ranks top in the first two cases and slightly less than the WKNN method in the last case.

Besides, Figure 12 also illustrates that, in the case of one missing dimension (6th AP outage), the CPs are less affected by different target dimensionality (*D* = 6 or *D* = 8) in terms of the three feature extraction methods. This could mainly be attributed to the fact the lost information of one dimension is more significant, whereas the number of reduced dimensions plays a less important role. Moreover, in terms of the LDE and LDA methods, both of their CPs are less than either of the WKNN or Kernel PCA method, but it is worth noting that normally LDE performs better than LDA without AP outages, however the LDA surpasses the LDE in the case of 6th AP outage, and comes close to it when the 4th and 24th APs are powered off. Aside from instability and weak robustness of the two methods in low dimension situations, it is mainly due to the fact that different APs contribute to different information entropy in an indoor positioning environment, which was well analyzed in our previous work [20].

**Figure 14.**
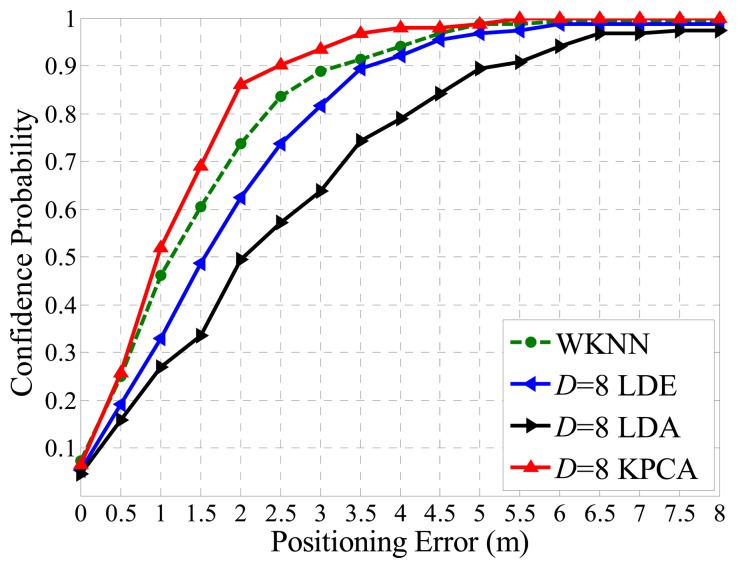
Positioning accuracies comparison in the noisy circumstance in S1 region.

For testing the robustness and noise tolerance of the proposed positioning system, we set it in an unstable and more noisy circumstance, where we take S1 region shown in Figure 7 as the interesting area with 152 reference points and leave doors and windows open, and in addition people walk around and RSS values are sampled only 1 time as a test point. The performance of proposed algorithm is better than the full dimensional WKNN fingerprinting method and other positioning systems as illustrated in Figure 14. Besides, it is worth noting that the situation of APs outage as shown in Figures 12 and 13 could be considered as an extreme noisy environment case, which may firmly prove the effectiveness of the proposed method as well.

**Figure 15.**
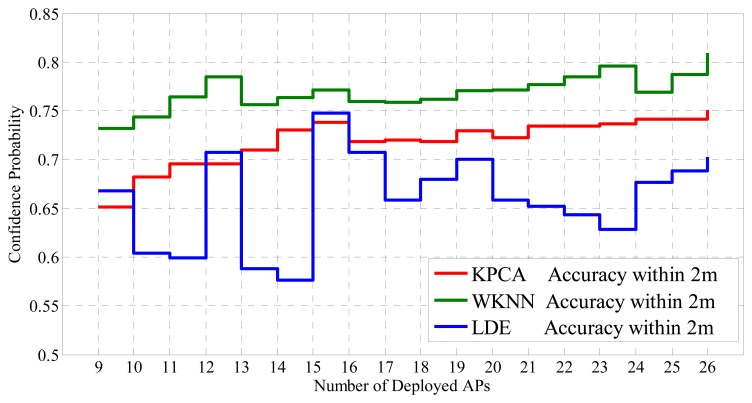
Performances of different positioning systems with different AP deployment under the condition that the positioning error distance is within 2 m and *D* = 8.

Moreover, environment dynamics including number of AP deployments and different sampling intervals are also taken into consideration. On the basis of ensuring all regions are being covered, performances of the proposed positioning system with different types of AP deployment are briefly evaluated as shown in Figure 15.

By and large, the confidence probability increases with the total number of deployed APs in terms of the WKNN method and the proposed system based on the KPCA method. However, the LDE shows outstanding positioning accuracy in some circumstances, e.g., fifteen APs are deployed in the building, though the instability of the method is obvious as well. Besides the reason that target dimension is unadjusted, the phenomenon can be partly attributed to the different discrimination of APs for different sample points, which is the reason that some researchers are concerned about AP selection schemes (to select most discriminating APs for positioning based on certain criterions, such as max mean, information entropy and joint entropy).

**Figure 16.**
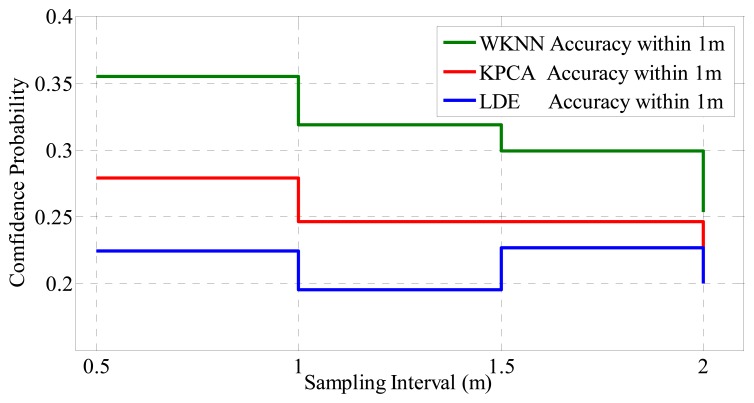
Performances of different positioning systems with different sampling density under the condition that the positioning error distance is within 2 meters.

In terms of the relationship between sampling density and the system performance, according to the experimental results shown in Figure 16, the confidence probability goes down slowly as the sampling interval increases (density decrease). Compared with the influence of APs deployment, the positioning accuracy is less affected by the sampling interval.

In sum, the Kernel PCA algorithm deployed in the proposed indoor positioning system is more capable of extracting the features of RSS with low dimensionality in an office environment, its robustness and generalization ability may provide higher positioning accuracy when dealing with asymmetric matching problem. The reduced dimension of the radio map may relieve the burden of the final online matching process, but it is undeniable that the computational complexity of the proposed method has increased in the previous feature extraction step. Specifically, the online computational complexity of the OG-SVM is O(C*n_sv_*), where *C* is the number of classes and *n_sv_* is the number of support vectors. The counterpart of KPCA is O(*dMN*), where *d* is the number of the (reduced) low dimensionality, *M* is the number of features (APs) and *N* is the number of reference points. Both of the LDE and LDA are O(*dM*). Besides, the computational complexity of WKNN method is O(*MN*). Therefore the computational complexity of the proposed positioning system is O(*dMN*) plus O(*dN*) and O(C*n_sv_*), so the other two systems share the same computational complexity, which is O(*dM*) plus O(*dN*). Compared with the two linear feature extraction methods (LDE and LDA), the proposed system underperforms others in terms of computational complexity due to the deployed kernel techniques. However, considering the contribution of dealing with unexpected AP outages and outstanding system robustness and stability, implementing the Kernel-PCA algorithm in the positioning system is still practical and effective.

## 5. Conclusion

In this paper, firstly we propose the SDC method for clustering the radio map based on both RSS in signal space and coordinates in physical space. Compared with traditional clustering algorithms, the proposed method is more flexible and without outlier problems and constraints. Experimental results show that the fingerprinting method based on the sub-radio maps clustered by SDC outperforms its counterparts based on the FCM and K-Means clustering algorithms. After being integrated with OG-SVM, the coarse positioning accuracy of the proposed method is also better than that of the other algorithms.

Then we deploy the Kernel PCA method for reducing the dimensionality of the radio map, thereby enhancing the robustness and solving the asymmetric matching problem when AP outages occur. It turns out that the proposed Kernel PCA performs better than the LDA and manifold LDE methods in terms of extracting the features of an indoor radio map.

**Figure 17.**
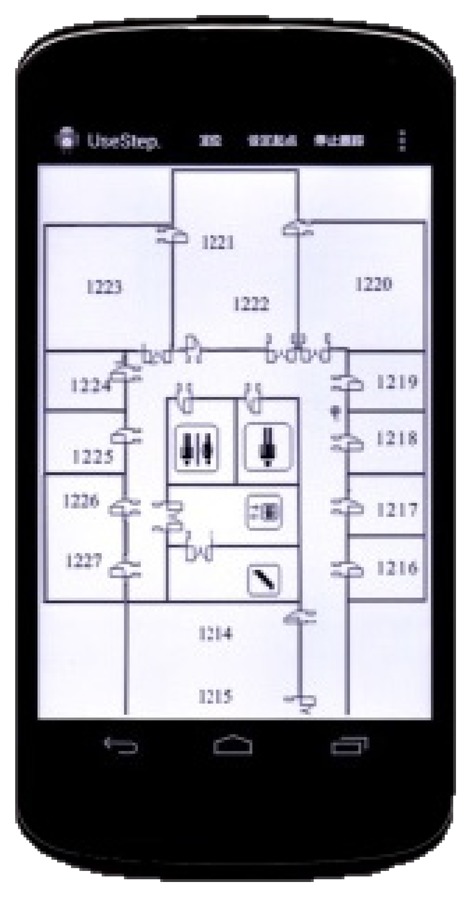
The proposed indoor positioning system running on Google Nexus 4.

In addition, the structure of the proposed indoor positioning system is well modularized and mainly designed for mobile computing. It consists of the offline phase and online phase, respectively. The off-line phase is in charge of the main data computing process with a powerful PC server. All the computed data and trained functions derived from the offline stage would be stored and applied in the online module for the real time positioning procedure. We have validated the feasibility and effectiveness of the proposed indoor positioning system, and implemented it based on the Android OS as shown in Figure 17. Besides APs selection, inertial navigation and other approaches for indoor positioning are also under further development. The section of performance analysis might not be described in great detail, but a lot of experimental and implemental works on localization have been done in this study. Our future works will also keenly focus on WLAN- and WSN-based indoor positioning systems, information from sensors such as gyroscopes, accelerometers, thermometers and barometers available within mobile terminals will be further researched and deployed in our positioning system.

## Figures and Tables

**Table 1.** Coarse Positioning performance of FCM method.

**Clusters**	**Number of RPs**	**Number of TPs Classified Correctly**
C1	146	145
C2	152	149
C3	132	121
C4	118	78
C5	100	81
C6	180	176

Classification accuracy: 90.58%		


**Table 2.** Coarse Positioning performance of K-Means method.

**Clusters**	**Number of RPs**	**Number of TPs Classified Correctly**
K1	216	216
K2	113	64
K3	155	130
K4	99	39
K5	145	133
K6	100	89

Classification accuracy: 81.04%		


**Table 3.** Coarse Positioning performance of SDC method.

**Clusters**	**Number of RPs**	**Number of TPs Classified Correctly**
S1	152	143
S2	136	132
S3	98	77
S4	108	96
S5	130	125
S6	204	204

Classification accuracy: 93.84%		

